# Improved inversion time (TI) scout sequence for late gadolinium enhancement MRI of patients with implantable cardiac devices

**DOI:** 10.1186/1532-429X-16-S1-P19

**Published:** 2014-01-16

**Authors:** Shams Rashid, Roderick H Tung, Kalyanam Shivkumar, J Paul Finn, Peng Hu

**Affiliations:** 1 Department of Radiological Sciences, University of California, Los Angeles, Los Angeles, California, USA; 2 Biomedical Physics Inter-Departmental Graduate Program, University of California, Los Angeles, Los Angeles, California, USA; 3 UCLA Cardiac Arrhythmia Center, UCLA Health System, Los Angeles, California, USA

## Background

Late gadolinium enhancement (LGE) cardiac MRI is the clinical gold standard for non-invasive characterization of myocardial scar [1]. The LGE technique is a contrast enhanced inversion recovery (IR) FLASH sequence, and requires a priori knowledge of the initial inversion time (TI) to produce optimal contrast between healthy myocardium and scar substrate. The TI is typically assessed using a Look-Locker based TI scout sequence. Recent advances [2,3] have enabled successful application of LGE MRI to patients with cardiac implantable devices at our institution. Presence of cardiac devices, such as implantable cardioverter defibrillators (ICD), gives rise to severe off-resonance in the myocardium. This produces banding artifacts in standard TI scout images which cannot be used to estimate the initial TI for LGE imaging. This can result in LGE images with poor contrast and delays in patient scanning. In this abstract, we present a modification to the standard TI scout sequence that prominently reduces artifacts in TI scout images.

## Methods

The TI scout sequence is an inversion prepared cine sequence with TrueFISP readout. Severe off-resonance from an ICD produces severe banding artifacts in TrueFISP images. In addition, in IR sequences, severe off-resonance can prevent inversion of spins in the myocardium, giving rise to hyper-intensity artifacts [2,3]. Recently, we demonstrated that a wideband (3.8 kHz) inversion pulse can overcome this off-resonance effect and invert off-resonant spins sufficiently, thereby eliminating hyper-intensity artifacts [2,3]. We modified the TI scout sequence by replacing the conventional inversion pulse (bandwidth: 1.1 kHz) with a wideband inversion pulse (spectral bandwidth: 3.8 kHz). We also replaced the TrueFISP readout with a FLASH readout. The modified sequence was tested on a group of T1 phantoms and one ICD patient.

## Results

**Figure 1:**
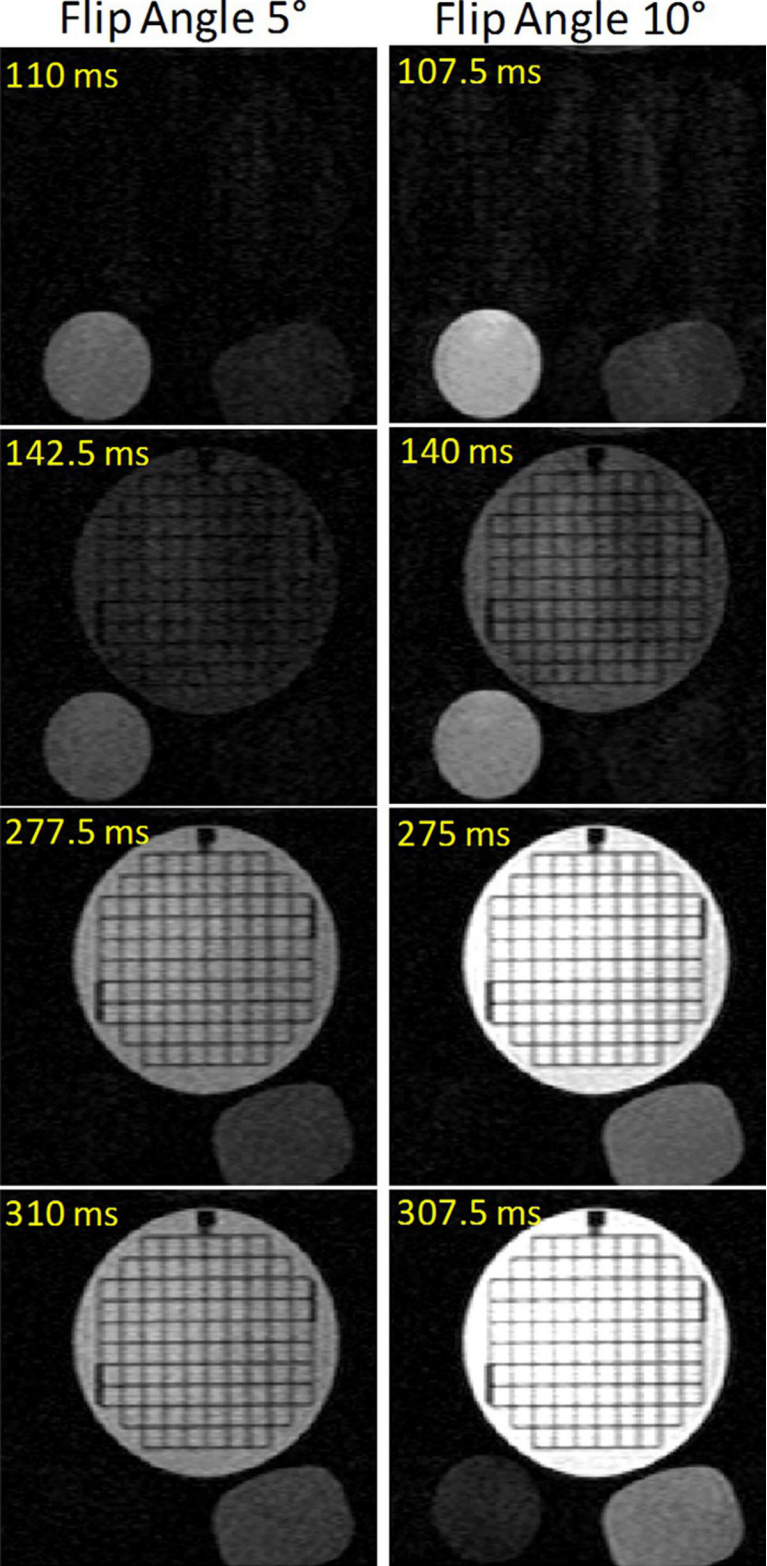
**Phantom results showing the modified TI scout sequence**. The large circular phantom has a T1 of 158 ms, and is optimally nulled by a TI of 105 ms. The small phantom on the bottom left has a T1 of 450 ms and is optimally nulled by a TI of 300 ms. The small phantom on the bottom right has a T1 of 210 ms and is optimally nulled by a TI of 140 ms. These images show that with a flip angle of 5°, the optimal TI is determined quite accurately. At a flip angle of 10°, low TIs are determined accurately, but higher TIs are underestimated, due to signal burn-off, as seen from the images in row 4.

**Figure 2:**
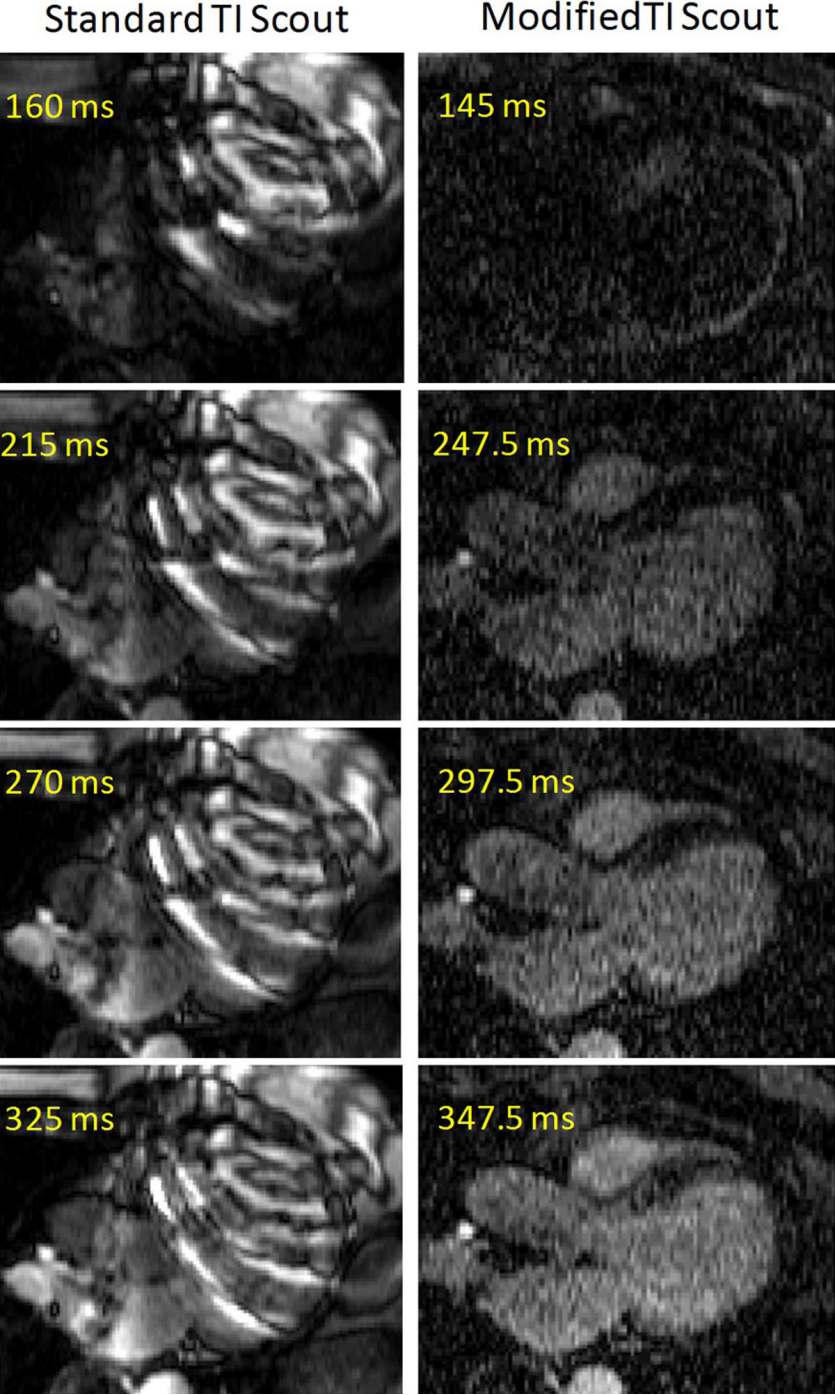
**Comparison of the standard TI scout sequence and the modified TI scout sequence in a patient with an ICD**. In the standard TI scout, off-resonance due to the ICD causes severe banding artifacts throughout the heart, rendering the scout images unusable for determination of optimal TI. In the modified TI scout sequence, the artifacts are eliminated, and the optimal TI can be determined.

Figure 1 shows phantom images of the modified TI scout sequence. The phantom results show that when a flip angle of 5° is used, the modified TI scout sequence can be used to determine the optimal TI quite accurately. At higher flip angles, larger TIs may be underestimated due to signal burn-off. Figure 2 demonstrates the banding artifacts produced when the standard TI scout sequence is used in an ICD patient. The modified TI scout sequence is able to remove these artifacts completely and allow effective determination of the optimal TI.

## Conclusions

We have modified the TI scout sequence by implementing a wideband inversion pulse and FLASH readout. Phantom results and patient studies demonstrate that the modified sequence has none of the image artifacts that occur with the standard sequence, and produces a reliable estimation of inversion time. We expect that the new TI scout sequence will lead to improved application of LGE MRI in patients with implantable cardiac devices.

## Funding

NIH 1R21 HL118533.
